# The Case for Animal Privacy in the Design of Technologically Supported Environments

**DOI:** 10.3389/fvets.2021.784794

**Published:** 2022-01-07

**Authors:** Patrizia Paci, Clara Mancini, Bashar Nuseibeh

**Affiliations:** ^1^School of Computing and Communications, The Open University, Milton Keynes, United Kingdom; ^2^Lero, Irish Software Research Centre, University of Limerick, Limerick, Ireland

**Keywords:** animal-computer interaction, animal privacy, privacy requirements, privacy aware design, multispecies interaction design

## Abstract

Privacy is an essential consideration when designing interactive systems for humans. However, at a time when interactive technologies are increasingly targeted at non-human animals and deployed within multispecies contexts, the question arises as to whether we should extend privacy considerations to other animals. To address this question, we revisited early scholarly work on privacy, which examines privacy dynamics in non-human animals (henceforth “animals”). Then, we analysed animal behaviour literature describing privacy-related behaviours in different species. We found that animals use a variety of separation and information management mechanisms, whose function is to secure their own and their assets' safety, as well as negotiate social interactions. In light of our findings, we question tacit assumptions and ordinary practises that involve human technology and that affect animal privacy. Finally, we draw implications for the design of interactive systems informed by animals' privacy requirements and, more broadly, for the development of privacy-aware multispecies interaction design.

## Introduction

Within interaction design literature, privacy has been an increasing concern, concomitantly with the increasing capabilities and pervasiveness of computing systems. The discourse on privacy has, so far, almost exclusively focussed on humans, disregarding the implications that interactive technology might have for other animals who might come into direct or indirect contact with it. Some of the authors who have most influenced the discourse on privacy within computing and interaction design had recognised early-on that privacy is not an exclusively human phenomenon and that animals show a need for privacy in various circumstances. In particular, starting from the analysis of territoriality, Westin (1) and other privacy scholars, described basic privacy-claiming and distance-setting mechanisms manifested in both human and non-human animals. Unfortunately, subsequent to this early work, the scholarly discourse on privacy has neglected to examine this fundamental phenomenon beyond the human species, which is reflected in a lack of consideration for the privacy of animals in the design of interactive systems.

With the increasing development and use of technology to manage animals in households, farms, zoos, research facilities and even wild environments, privacy considerations when designing such systems have become ever more important. For example, farmers who monitor their animals electronically face exposure to cyberbreaches and recognise the importance of data protection mechanisms (2). Typically, the motivation for developing cyber security and privacy protection mechanisms in animal contexts is still a need to protect data “owned” by humans, rather than a concern for the privacy of the animals themselves. However, cyberbreaches may indeed have a serious impact on animals' lives. For example, it has recently been brought to the public's attention that data captured from GPS collars fitted on protected wildlife can be intercepted, exposing tagged animals to the attack of cyberpoachers; in response, some have called for the development of security systems that cannot be easily hacked by poachers, so that the life of the animals, rather than the data “owned” by researchers, can be protected (3).

But, in a world where animals are constantly exposed to human technologies, are privacy concerns only limited to data security and bodily safety in the context of illegal practises or do animals have other privacy needs too? What privacy dynamics, if any, do animals manifest that might need to be taken into account when designing interactive systems which may affect them, or which are specifically designed for them? How might animal privacy be considered when designing technologically supported environments? To address these questions, we searched a wide range of literature for sources that might discuss privacy-related behaviour in animals to understand the existing discourse on the topic. We found that related scholarly works are sparse across domains and that the notion of animal privacy is under-defined and under-researched. Hence, Animal-Computer Interaction researchers navigate uncharted waters when undertaking the challenge of designing technologically supported environments that might require consideration for animals' privacy needs.

To better understand animal privacy, we analysed animal behaviour literature that could illuminate what privacy-related processes are manifest among animals. We based our analysis on the definition of privacy mechanisms provided by early literature on privacy and found that animals use a variety of privacy-related mechanisms, whose function is to secure their own and their assets' safety, as well as negotiate complex social interactions. In light of our findings, we questioned tacit assumptions and ordinary practises that involve human technology and that affect animal privacy. We did so by extending the notion of privacy to animals and discuss how animal-centred interactive systems could consider animals' privacy requirements.

## Background

### Animals' Privacy in Interactive Systems: An Emerging Design Requirement

In recent times, the Animal-Computer Interaction (ACI) (4) community has started investigating the privacy concerns of pet guardians when they use wearable devices to monitor their pet activities. In particular, van der Linden et al. (5) investigated the extent to which dog tracker users are aware of, and guard themselves from, potential risks to their own privacy that may come from a data breach in the tracking system. The authors concluded that dog carers are primarily concerned about physical safety consequences (e.g., dogs being stolen, and houses being burgled) since these devices can reveal data about the carer's habits and caregiving practises. In another study, the same authors describe potential personal threats to humans derived from the use of animal GPS collars. For example, these risks might occur if dog walkers were to share their habitual routes online through tracker device applications, or if malicious individuals were to breach pet location data logged into the device in order to commit pet theft (6). The authors refer to the theory of the extended self (7) to explain pet-owner relationship in relation to privacy and claim that strong animal-human bonds result in greater risks of privacy and security breaches enabled by data from animal wearables. The findings of these studies indeed show implications for the design of “privacy-respectful” pet wearables and highlight the need to introduce privacy and security safeguards to prevent data breaches. In this work, animal privacy is investigated as an extension of human privacy, whereby what is at stake is the safety and security of pet guardians' property and relationship with their pets. But is privacy just a human concern or is it important also from animals' perspective?

For example, like other animals, dogs tend to avoid both actual and perceived threats. Given the probability that being separated from their guardian is perceived by many dogs as a threat, would they not want to protect themselves from such potential harm if they were aware that the wearable system attached to their body could be breached with ill intent? Unbeknown to them, technological interventions can expose animals to serious threats which they would arguably want to escape if they were able to perceive the danger they were in.

In response to the proliferation of humans' technologically mediated intrusions upon other animals, Mills (8) questioned the ethical legitimacy of practises such as physically entering animals' territories or placing cameras into their hiding places in order to film them. Mills' argument was grounded in the observation that animals demonstrate a want for physical separation and withdrawal. At the time, Mills' argument found opposition from various quarters, including animal welfare and conservation organisations, who defended the value of using filming technology to increase people's awareness of and empathy for animals. Notwithstanding the educational value of these interventions, one might question the assumption that humans are best placed to make this kind of risk-benefit assessments, instead of (somehow) allowing the main stakeholders to do so. In this regard, Haratym (9) pointed out how Mills' argument was no different from that famously made by Warren and Brandeis (10) with regards to the use of technological devices to record and store detailed information on individuals which can be later disseminated to the public. While she recognised that avoiding any interference with their private sphere may be very difficult, Haratym argued that animals manifest the need for separation from others (i.e., privacy) and calls for the recognition of their “right to be let alone” (9, 10).

### Animals' Privacy in the Early Privacy Literature: A More-Than-Human Phenomenon

While the notion of human privacy has significantly developed over time to include many dimensions such as personal, intimate, and social privacy, the phenomenon of animal privacy has received very little attention. The only existing conceptualisations are those of early privacy scholars, who theorise the phenomenon at a more fundamental level, mainly to explain the origin of privacy in humans. In his seminal work on Privacy and Freedom, Westin (1) made direct reference to Ardrey (11)'s writings on territoriality to argue that humans' need for privacy is likely rooted in our animal origins, and that humans and animals share a number of basic privacy-claiming mechanisms. Territoriality would be one such mechanism, whereby an organism “lays private claim to an area of land, water or air and defends it against intrusion by members of its own species,” “to ensure propagation of the species by regulating density to available resources” and “to promote individual well-being and small-group intimacy” (1, p. 26). Humans and other mammals would also share distance-setting mechanisms that exploit sensory (olfactory, acoustic, visual, tactile) information to maintain personal, intimate and social boundaries in interpersonal relationships (1, p. 26). Citing Calhoun (12)'s work on rats' behaviour, Westin highlighted how overpopulation without the possibility of maintaining privacy boundaries impairs animals' ability to preserve social organisation, leading to serious disfunctions, such as chronic stress, constant fighting or sexual sadism. On the other hand, when afforded the opportunity to maintain privacy boundaries, social animals still seek the stimulation of encounters among their own species. Thus, privacy boundaries enable animals to maintain functional social interactions while protecting individuals from others' interference when they need to access resources that are necessary for their survival.

Later, Klopfer and Rubenstein (13) articulated the biological basis of privacy in economic terms. The authors distinguished two types of privacy that animals would manifest to varying degrees and at different times depending on their level of sociality: physical separation and information management. While territoriality would afford animals physical separation on a stable basis, various forms of concealment would afford them temporary withdrawal (e.g., when giving birth or hiding from predators). Social animals would also achieve privacy by preventing others from acquiring complete and accurate information about them or their intentions, which could be used to access resources. In this regard, the evolutionary transformation (ritualization) of behaviour patterns into communicative signals whose form is not associated with the animal's motivational states would enable an individual to withhold information, thus attaining a measure of privacy that might give them a competitive advantage (e.g., in order to deter a competitor, an animal might signal that they are about to attack, when in fact they have no intention of doing so). Since maintaining privacy has costs (e.g., having to keep guard, losing social input) as well as benefits, and social interaction has benefits as well as costs, animal populations would seek a cost/benefit equilibrium that is optimal for their fitness. Like Westin, Klopfer and Rubenstein noted how the ability to maintain privacy is essential to animals' fitness, and how privacy violations (e.g., territorial intrusions) or living conditions that prevent animals from maintaining privacy (e.g., in captivity) lead to behavioural and physiological dysfunctions. Additionally, Klopfer and Rubenstein's analysis of privacy as a cross-species phenomenon manifested through species-specific mechanisms parallels Altman's (14) influential work on human privacy, in which he describes the phenomenon as a cultural universal manifesting through culture-specific mechanisms.

Like Altman, Hirshleifer (15) talked about privacy as the means to dynamically achieve autonomy within society but, unlike Altman, Hirshleifer's model accounts for the biological as well as the cultural evolution of privacy. His analysis of the origin and function of privacy classifies the main structures of sociality in all animals based on three principles: dominance, communal sharing and private rights. The dominance principle would prevail where resources are dispersed and threats ubiquitous, and where there are advantages to being dominant (e.g., having privileged access to resources) but also to being subordinate (e.g., receiving protection). The communal sharing principle would prevail where acquiring resources (e.g., food) or safeguarding common goods (e.g., genes) requires cooperation and mutual support. The private rights principle would manifest through territoriality (over e.g., land, food sources, sexual mates), and would prevail where resources are fixed in place and stable, and where social organisation and role diversification can increase fitness and prosperity. For Hirshleifer, each structure has evolved in a particular ecological context where it provided a survival advantage, but all structures would manifest themselves in different circumstances. Critically, Hirshleifer points out how each social structure could only persist if associated with what he terms an ingrained supporting ethics, that is an evolved ethics that most members of society accept and live by out of reciprocity, thus ensuring individuals' compliance (15). With regards to territoriality, the ethics supporting privacy behaviours would manifest in the outsider's reluctance to intrude (other than surreptitiously) and in the defensive belligerence of the proprietor aimed at protecting their assets. In other words, the insistence on one's own rights and the willingness to concede the same right to others would be the two sides of the same “ethic coin” enabling territoriality to function as a social organisation principle.

To summarise, according to these early scholars, the need for privacy is a biological universal, whose purposes include preserving *personal safety*, ensuring *access to resources* and managing *social relations*. The distance-setting mechanisms through which these purposes are achieved include different forms of *physical separation* (e.g., territoriality, physical concealment) and *information management* (e.g., witholding, deception). Furthermore, animals living within a social ecosystem abide by the ethics that legitimise these mechanisms. The aim of our study was to find evidence of privacy behaviour in animals, the purpose that the behaviour might have, the mechanisms by which that purpose might be pursued, and the underpinning ingrained ethics.

## The Study: Exploring Research on Animal Privacy

We reviewed a wide range of literature reporting on ethological and behavioural experimental studies that had investigated the behaviour of different animal species. As mentioned above, we sought to identify some of animals' *physical separation* and *information management* mechanisms (13), and understand their function in context in relation to *personal safety, access to resources* and *social relations* (1). We were also interested in any expressions of the evolved supporting ethics that might compel individuals to respect others' privacy boundaries, in turn enabling them to enjoy the same benefits (15). Our aim was to search for compelling examples of animals' manifest privacy-related behaviours that could help us frame the issue of animal privacy with a view to informing the design of interactive systems involving more-than-human stakeholders.

### Generation and Analysis of the Dataset

We performed our search for literary sources using data drawn from three major scientific knowledge databases containing publication records: Microsoft Academic Graph (MAG), Google Scholar (GS), and Web of Science (WoS). We extracted the data from the datasets using an Elasticsearch (ES) index (16), which firstly organised the metadata of each paper in terms of title, abstract, relevant topics, and other features, and then provided an engine for querying such data. To obtain our first set of potentially relevant papers, we queried the produced ES index looking for papers whose titles, abstracts, and whole texts contained the keywords “animal” and “privacy.” We used general searching criteria because we wanted to explore whether in ethology and experimental behavioural literature privacy had ever been investigated as a distinctive animal mechanism. Through this process we obtained 928, 189, and 97 results, respectively from MAG, GS, and WoS. However, after reading titles and abstracts of each obtained result, we found that in most of the sources including the words “privacy” and “animal,” these were entirely unrelated to each other (e.g., medical experiments using laboratory animals and discussing the privacy of human patients). Any abstract referring to some kind of animal behaviour led to reading the whole paper in search of connexions with the notion of privacy (which being one of our keywords had to be present somewhere in the paper). Finally, a paper was selected for our dataset if it described an animal behaviour that expressed a *physical separation* or *information management* mechanism of some kind. More specifically, our selection was informed by the following criteria:

MAIN CRITERION - is an individual performing some kind of *distance-setting behaviour*?

SUB-CRITERIA (SC) SC1 - is the behaviour establishing *some form of physical separation/proximity*? i. *what kind* of physical separation/proximity is the behaviour achieving? ii. *via what means* is the separation/proximity achieved? iii. *what function* is the separation/proximity performing?

SC2 - is the behaviour *concealing/disclosing some kind of information*? i. *what kind* of information is the behaviour concealing/disclosing? ii. *via what means* is the concealment/disclosure achieved? iii. *what function* is the concealment/disclosure performing?

These inclusion criteria were based on the definition of privacy mechanisms found in the early literature on privacy, to control against bias in our selection process. This approach was informed by Stern and MacArthur (17)'s guidelines for screening sources against inclusion and exclusion criteria and allowed us to select a first set of papers (*n* = 5). Then, we searched the citation lists of each paper to find further sources following a snowballing procedure, as described by Wohlin (18).

In total we analysed 22 scientific papers published, between 1966 and 2010, in the following venues: Animal Behaviour (*n* = 7), Journal of Perinatal Education (*n* = 1), Behaviour (*n* = 1), Zoo Biology (*n* = 1), Journal of Experimental Animal Science (*n* = 1), Obstetrics and Gynaecology (*n* = 1), African Journal of Ecology (*n* = 1), Bioacoustics (*n* = 1), Ethology, Behavioural Ecology (*n* = 1), Animal Cognition (*n* = 1), Journal of Comparative Psychology (*n* = 1), The American Naturalist (*n* = 1), Communicative and Integrative Biology (*n* = 1), Journal of Fish Biology (*n* = 1), Frontiers in Zoology (*n* = 1), and Cambridge University Press's Animal Communication Networks article collection (*n* = 1). We analysed animal behaviours reported by these sources to understand (1) what *physical separation* or *information management* mechanisms they might express in different contexts and (2) for what function (*safety, resources, relations*).

Although we used general keywords to explore the extent to which privacy is explicitly linked to animals, this approach might have limited species and taxa's representation in the article sample. For example, we did not find papers concerning amphibia and reptiles, or many other social species where we might have expected privacy to play a role. This does not mean that no such papers exist and the fact that we did not find any may well reflect the limitation of our approach. Nevertheless, we thought it important to maintain the systematicity of our surveying approach. Furthermore, the fact that no papers focussing on other taxa and species emerged from our general search is in itself a result, suggesting that the topic of privacy in animals is still unexplored both within animal behaviour research and animal-computer interaction research. Shedding light on this blind spot was a key aim of our paper.

### Data Analysis

We analysed the text of the selected papers as follows: on first reading, pertinent excerpts of text reporting relevant animals' abilities and behaviours were extrapolated. Then, each excerpt was re-read for confirmation according to the inclusion criteria expressing *physical separation* mechanismsor *information management* mechanisms. We identified a wide range of privacy-related behaviours across various species and taxa, and then we searched for common themes to analyse their functions. In the next section, we discuss the functions and modalities of the privacy-related behaviours that we identified.

## Findings

We found that animals express a wide range of privacy-related behaviours, which constitute different forms of physical separation and information management, to ensure their and their offsprings' safety, protect their assets, gain access to mates, and manage social interactions and relations in different contexts.

### Privacy and Physical Distantiation

Animals' need for privacy is evidenced by a range of behaviours that provide safety for vulnerable individuals, protection of resources, access to mates, and that enable individuals to manage their social interactions by including, excluding or deceiving others.

#### Personal Safety, Social Space and Intimacy

One of the most obvious functions of privacy is to protect oneself from potential predators, which many animals achieve by physically concealing themselves on particular occasions. For example, Lothian (19) argues that various mammal species seek quiet and secluded spots to hide during labour in order to protect themselves when they are most vulnerable and to deliver their offspring away from danger. The author reports on Newton et al.'s (20) conducted on pregnant laboratory mice who were subjected to distressing environmental conditions. Lothian concludes that a “lack of privacy” induced the pregnant females to interrupt early labour to move away from the disturbance (19). In nature, this “self-retreating” behaviour might happen for protection against predators and competitors; the latter might include males who do not belong to a female's social group and who might kill her offspring, so that she will go into oestrous again and they will be able to fecundate her to the advantage of their own genes. For example, in African lions (*Panthera leo*), among whom infanticide occurs, lionesses separate from their group to give birth and nurse their young, and only reunite with the group when the cubs are 4–8 weeks old (21).

In some species, even when the presence of others does not pose an obvious danger to one's safety, individuals who live in close proximity to conspecific occasionally seek periods of seclusion, where interaction with other cohabitants is avoided. In a study involving rhesus monkeys (*Macaca mulatta*) caged in pairs in laboratory settings, individuals spent some time out of their cage-mate's sight, when their enclosure was provided with a separating panel; being able to temporarily seclude themselves in a dyadic social context seemed to help the monkeys get along better with each other (22).

Voluntary separation from one's cohabitants may also be sought to provide the opportunity for exclusive interaction with specific individuals at particular times, as observed in bottlenose dolphins (*Tursiops truncatus*). Mello et al. (23) studied the behaviour of three dams (female dolphins) living in artificial pools, who avoided contact with members of the group before and for some time after giving birth to their calves. In particular, they proactively sought solitude to nurse, suggesting that privacy facilitates the bonding with the calf and the synchronisation of the swimming pattern of mother and calf during nursing (23). As mentioned earlier, in various mammals, giving birth and nursing is done privately in burrows or caves; since dolphins live in open aquatic environments that do not offer dens and do not afford physical seclusion, dams' avoiding contact with others might be a privacy behaviour that has evolved to replace self-concealment strategies during mother-offspring caregiving.

#### Protecting Assets

Protecting acquired resources is vital for many animals, to which end physical concealment is often used to protect assets that are essential for one's survival. Various mammal and bird species store food in order to have access to a stable supply throughout scarcity seasons. Caching (storing covertly) is a strategy used to protect food from foraging competitors. For example, naturally foraging grey squirrels (*Sciurus carolinensis*) adopt food caching strategies that reduce the risk of giving away their stockpiles' locations to pilferers (sneak thieves). In the presence of potential conspecific pilferers, individuals implement “secretive” tactics, such as orienting themselves away from observers when they dig and spacing caches more widely (24). Another tactic is decreasing caching behaviour when individuals perceive the presence of observers. This happens both with conspecifics (in this case, other squirrels) and with heterospecifics (e.g., blue jays) (25).

Some birds use deceptive tactics to conceal food from competitors. For example, rooks (*Corvus frugilegus*) cache food cautiously hiding the activity when conspecifics are around, such as caching in long grass where their activity is less likely to be observed [personal observation of Emery and Clayton, in: (26)]. However, they do not adopt the same prudence in the presence of other rooks who are also engaged in caching [(27), cited in: (26)], as though they were “confident” that other rooks focused on the same activity would not be interested in pilfering.

There is some evidence that storing tactics develop in specific circumstances following specific events. For example, in a two-experiment laboratory setting, Preston and Jacobs (28) observed wild-caught Merriam's kangaroo rats (*Dipodomys merriami*) overtly storing seeds for later use, in the presence of both conspecifics and heterospecifics (in this case, chisel-toothed kangaroo rats). However, after they experienced pilferage, they changed caching sites choosing more out-of-sight areas, even though these new areas had not been the spots initially preferred (28).

Similarly, western scrub-jays (*Aphelocoma californica*) who suffered pilfering of their caches in the past, unlike naïve individuals, tended to cache in new sites, out-of-view sites or shaded sites when observed by competitors [from Emery and Clayton (29), cited in (26)] or maximise the distance from an observer when this could not be left out of sight [(30), cited in: (26)]. The birds also repeatedly moved specific caches that were hidden while observers were watching, possibly to confuse them, and recached items as soon as they were given a private moment from others (26). However, when scrub-jays do not see competitors around, they show no preference between shady and well-lit sites [(30), cited in: (26)], suggesting that experience might play a role in their performance of privacy behaviours.

Interestingly, scrub-jay mates defend each other's caches from conspecific pilferers, demonstrating a sharing of knowledge about caches between the pair [(31), cited in: (26)]. Similarly, in ravens (*Corvus corax*), who are used to feeding in non-kin groups (congregations) but move away from the food source to cache food when other ravens are feeding on the same source, mating pairs cache together and therefore share the location of caches with their mate (28). Thus, while concealing caches from other ravens is probably a strategy to avoid pilferage, disclosing food location to partners may have various functions such as mate bonding and pair breeding success.

#### Securing Access to Mates

One important relation many animals have is that which they have with mates; mates also constitute fundamental genetic resources. To gain privileged access to mates and secure reproductive success, some species employ tacticts such as concealment and deception. In laboratory settings, male guppies (*Poecilia reticulata*) and three-spined sticklebacks (*Gasterosteus aculeatus*)-(two species of fish)-move to concealed areas of the tank to court a female, if rivals are around. This is hypothesised to avoid interference from other males and to increase the chances of copulation with the targeted female (32, 33). Experiments specifically showed that when male sticklebacks do not have the possibility of using concealed areas, they tend to court less (i.e., they perform fewer of the zigzag tail movements typical of courtship) and instead direct part of their attention to attacking rivals. It could also be that they avoid engaging in an activity that would reduce their alertness where they might be exposed to predators and fall easy prey (33). On the other hand, when they are given the opportunity of using a concealed area, they spend more time there, if a female is available.

These findings are consistent with those from another study that investigated physical concealment in sticklebacks when they want to intrude one another's mating nest. Intrusion into someone else's territory is potentially hazardous, but it is motivated by the potential advantage of acquiring a mate. In this species, external fertilisation occurs, whereby males prepare a nest where females lay their unfertilized eggs for males to fertilise, following a successful courtship. One reproductive strategy of three-spined sticklebacks males is to breach into the nest of a resident male who is courting a visiting female. To this end, sneakers disguise themselves assuming a drab coloration, which renders them harder to detect in silty water and allows them to move close to the eggs to eventually fertilise them before the nest owner has a chance to do so (34). This deceptive behaviour, used by the perpetrator to mask his intention, might stimulate resident males to want to hide their courtship from other sneakers in order to avoid nest intrusion.

Male guppies use deception to improve their mating chances, by decreasing their courtship of females they had previously targeted, if mating competitors are around and there is no possibility to hide (35). It is hypothesised that in this way they disguise their interest in order to trigger a copy effect, thus causing other males to also lose interest in the targeted female. It is the same for male Atlantic mollies (*Poecilia mexicana*), who overtly direct their mating interest toward a non-preferred female when other males are on sight. This seems to have the function of misleading other males about which female one prefers for mating before proper courting is initiated (36). These behaviours can be interpreted as concealment of real interest and intention (rather than concealment of the courting and mating activity itself), in order to gain privileged access to desired social relations and resources.

### Privacy and Vocal Communication

Aside from being expressed through physical distantiation, animals' need for privacy is also evident in and achieved through different vocal communication modalities aimed at safely maintaining relevant social relations remotely or in intimate situations.

#### Safety and Connectedness in Remote Social Interactions

Lions use vocalisations to negotiate between the need for self-preservation and the need for group living (37); they conceal or disclose their presence and identity depending on their momentary need for safety or for contacting pride members. For example, when they are in their territory, the females and males within a group roar to advertise territorial boundaries, to contact pride-mates who are away from it, and to attract sexual mates. However, when they are outside of their territory and away from their own pride, they remain silent (37); while mothers modulate their roaring depending on whether they are alone or in group to avoid the risk of attracting extra-pride males who might commit infanticide (38). Lone lions who do not have a pride usually make contact calls with other lone individuals, disclosing their presence and identity for the purpose of creating and maintaining (some sort of) association for hunting purposes. However, if they are in the territory of rival individuals who can threaten them, they stay silent keeping secret their presence. Low signalling rates or suppression of calls avoid giving away one's position, identity, and group membership to unwelcome listeners. Individuals may even prefer to remain isolated rather than communicate with potential mates, if there is a risk that they might give away their presence to threatening competitors.

#### Safety and Connectedness in Intimate Social Interactions

Observations in the wild revealed that female chimpanzees (*Pan troglodytes*) mate overtly or covertly depending on their and their partners' rank. They also disclose or conceal copulating events by modulating pitch and loudness of copulation calls when mating with dominant or low-rank males. This seems to be related to retention of “social status rules,” in order to avoid aggressive behaviours from high-ranking individuals (39). Specifically, when low-ranking copulating females are near high-ranking females, they produce fewer copulating calls, especially if copulation is with high-ranking males. This is probably to diminish the risk of aggression from high-ranking females, which has both social and safety purposes. Disguising communication during mating activities have been observed in fish, mammals, and bird taxa as well. For example, during the breeding season, male blackbirds (*Turdus merula*) sing quiet twitters directed at specific females, while female blackbirds emit quiet copulation trills to prevent detection from neighbouring conspecifics. These so called “quiet songs” have high frequency features that restrict the distance of transmission and can be directed toward particular individuals, so they are emitted by birds during close range and direct interactions. The phenomenon of quiet songs in songbirds is poorly explored (40) but these are good candidates for private signals. They are performed during sensitive activities in the breeding season and individuals use them to control who has access to the “arousal” information that foreruns copulation. Hence, they reveal an individual's intention to breed with the targeted mate, but conceal it from neighbours who might disrupt a pair's mating activities.

#### Selective Communication

Studies show that odontocetes (i.e., marine mammals like dolphins and whales) may be able to privately address information to specific individuals, potentially strengthening group bonding. In particular, dolphins' clicks (a type of call) and killer whales' high frequency calls are highly directional signals that can be potentially addressed at individuals ahead of the caller. These restricted range transmissions allow the signallers to share information such as their identity, location and direction of movement with specific receivers (41) while withholding it from a generic audience (42).

African elephants (*Loxodonta africana*) provide another example of private communication within a group to maintain social relations. These animals are able to discriminate familiar and unfamiliar calls, the former being used for maintaining contact and coordinating with members of a same group across long distances. For example, there is evidence that calls from family members are significantly responded to and followed by approaches to the area from which the call had originated (43). The author demonstrated that vocalisation brings information about presence and identity of callers and that this is shared for bonding and reunification purposes. However, although familiar calls are addressed at one's group members, the loud nature of long-distance vocalisations and the propagation medium makes this type of communication prone to interception by listeners and does not allow the caller to stop unwanted listeners from eavesdropping (43). Although from our analysis we did not find studies on “secretive” communication in elephants, this cannot be excluded if we consider what happens in other species. For example, various seal species (i.e., *Leptonychotes weddelli*) use colony-specific calls and dialects to recognise their members and possibly deliver private messages to specific groups or individuals among colony members (42). However, as with elephants, these loud calls are audible from a great distance and every individual who knows “the code” could be a receiver able to interpret the message.

## Discussion

### Animal Privacy as Social Organistion Principle

As theorised by early privacy scholars, our peruse of literary sources shows that, far from being a uniquely human phenomenon, privacy is indeed a concern of other animals too. Whether privacy-related behaviours are conscious or unconscious, learnt by individuals or genetically inherited, they evidence the value that privacy boundaries have for animals. Indeed, at least with regards to the species discussed in the literature, animals go to significant lengths to implement a range of distance-setting mechanisms to modulate the boundaries of their interaction with others, to include or exclude different individuals at different times (as proposed by Westin), either through physical separation or through information management (as specified by Klopfer and Rubenstein).

*Physical separation mechanisms*, such as self-retreating behaviour, may have the function of protecting one's own safety and the safety of close relations (e.g., possibly, those who carry one's own genes) at moments of particular vulnerability, as in the case of mice or lionesses who sought separation from their social group to give birth and nurse their newborn; or in the case of sticklebacks who prefer to court in secluded areas to avoid exposing themselves to potential predators when their attention is focussed on courting procedures. But, separation *from* others does not simply exclude intruders when their presence might be dangerous or not relevant, it also creates the opportunity to develop intimate relations by allowing exclusive interaction with those one separates *with*, as in the case of the dams who separated from their group to bond with their calf and of courting stickleback pairs. Furthermore, separation may provide temporary relief from the social pressure of having to live in close proximity with someone, as was the case with the rhesus monkey pair living in a lab.

Physical separation may concern the protection of resources as well as the protection of individuals' safety, as was the case with grey squirrels, kangoroo rats, rooks and scrub-jays, who used various distancing tacticts to prevent pilfering of their food caches. This included orienting themselves away from observers when caching (squirrels) or caching out of sight (kangaroo rats); it also included the use of deception, such as hiding the very activity of caching (rooks) and re-caching when not seen (scrub-jays), often as a result of having experienced pilfering (kangaroo rats, scrub-jays).

Of course, physically hiding food or the activity of caching food is also an *information management mechanism*; not only is hiding a caching activity a way of preventing others from acquiring information about it, but re-caching food is also a way of providing false information to derail a potential competitor. This kind of information management is particularly evident in the deception tactics used by some animals to attain reproductive success, as in the case of sticklebacks who camouflage to render themselves invisible to rivals and sneak into their nests to surrepotitiusly fertilise females' eggs; or as in the case of guppies who seemingly hide their intention to pursue a female in order not to stimulate rivals' own interest in the same female. Where information management mechanisms are particularly evident is in the case of vocal communication. Related literature provides examples of animals using overt vocalisations to signal their presence to others when it is safe to do so, as with lions when they are in their territory, while refraining from vocalising when it is not safe, as lions do when they traverse others' territories or when stronger rivals might be in the vicinity. Information management also takes place during mating, as with female chimpanzees who modulate their mating calls depending on the rank of the males they are mating with and the rank of females in the vicinity to avoid repercussions for their social transgressions; and as with blackbirds who exchange quiet songs to establish an exclusive and intimate communication channel with their mate. Additionally, the use of directional calls by orcas and of colony-specific dialects by seals is a way of selectively managing the information these animals share with others.

Furthermore, literature provides examples of what Hirshleifer calls ingrained supporting ethics related to different social regimes and corresponding privacy behaviour. For example, the case of subordinated female chimpanzees who subdue their copulation calls when they mate with higher ranking males in the vicinity of higher ranking females shows awareness of a *dominant-subordination ethics* whereby an individual acknowledges that dominant members of the group have priority over certain privileges and that any insubordination must be discreet if they are not to lose their standing in (and protection from) the group. Similarly, the ravens' caching behaviour exemplifies a *resource acquisition-sharing ethics* whereby animals aim to acquire and protect resources for themselves but are willing to share them where the sharing supports a common good, such as reproduction. This also might suggest that ingrained supporting ethics underpin at least some altruistic behaviours, ensuring reciprocal privacy and related benefits. Finally, the behaviour of dolphins and lions exemplifies compliance with or awareness of a *territorial ethics*; in particular, the dolphins who live within the close confines of a tank respect the spatial boundary that dams set by distancing themselves from the rest of the group to bond with their newborn; and lions refrain from vocalising when they know that they are crossing other lions' territories to avoid advertising their presence. In all these cases, animals show awareness of the ethics that underpin the social regimes of which they are part and either comply (raven pairs, dolphins) or, if they transgress, they do so surreptitiously (chimpanzees, lions).

Table 1 summarises the distance-setting mechanisms, as well as their manifestations and purposes, that we found in the literature.

**Table 1:** Species showing privacy-related behaviours.

	**Species**	**Paper**
**Physical separation mechanisms**		
Hiding during labour to protect themselves and offspring from potential danger	Various mammals, E.g.: Mice African lions	Lothian (19) Newton et al. (20) Rudnai (21)
Avoiding contacts with group members to nurse	Bottlenose dolphins	Mello et al. (23)
Spending time away from mates' sight	Rhesus monkeys	Reinhardt and Reinhardt (22)
Orienting themselves away from observers to cache food	Squirrels	Leaver et al. (24)
Caching in out-of-sight sites from observers or after experiencing pilferage	Kangaroo rats Western scrub-jays	Preston and Jacobs (28) Dally et al. (26)
Hiding caching activities from observers	Rooks Ravens	Dally et al. (26) Heinrich and Pepper (32)
Courting females in concealed areas	Guppies Sticklebacks	Hibler and Houde (44) Dzieweczynski and Rowland (44)
**Information management mechanisms**		
Comouflage to sneak into others' nest surrepotitiusly	Three-spined sticklebacks	Vlieger and Candolin (34)
Disguise interest to deceive competitors in mating	Guppies Atlantic mollies	Makowicz et al. (35) Ziege et al. (36)
Suppressing calls to hide presence, position and identity	African lions	Grinnell and McComb (37)
Mating overtly or covertly depending on partners' rank	Chimpanzees	Townsend and Zuberbuhler (39)
Directing ‘quiet’ calls and trills to potential mates	Blackbirds	Dabelsteen et al. (40)
Advertising territorial boundaries	African lions	Grinnell and McComb (37)
Sharing caching locations with mating partners	Ravens	Heinrich and Pepper (44)
Selective transmission of vocal information	Dolphins, killer whales	Janik and Slater (41) Janik (42)
Group-specific calls and dialects for private communications	African elephants Weddell seals	McComb et al. (43) Janik (42)

In short, it seems evident that privacy, in the forms and via the mechanisms discussed above, underpin animals' social organisation and fundamental biological functions. We propose that this has important implications for human-animal co-habitation generally and for the design of technologically supported environments more specifically.

### Animal Privacy as a Design Principle

Loss or reduction of natural habitats and territories is a recurring issue in human-made environments, which links the problems of privacy boundaries and human-animal co-habitation together. Since hunter-gatherers abandoned their nomadic lifestyle to cultivate the land around 10,000 BC, humans have increasingly converted natural environments into anthropogenic habitats (45), settling and expanding to accommodate the needs of an increasing human population. However, settlements take over land already inhabited by wildlife who shelter, forage, and reproduce in dens, vegetation, and waterways. When roads and edifices are built, animals are either killed or displaced (a practise commonly described as “expropriation” or “occupation” when inflicted upon humans). With the exception of endangered and law-protected wildlife, there is little attention to the destiny of displaced individuals, who end up living in smaller and fragmented intra-urban natural habitats or attempt to repopulate their former spaces now occupied by humans (46). However, many urban environments do not provide the spaces and resources many animals need to live in an ecologically equilibrate way, including the ability to implement and observe appropriate distance-setting mechanisms, particularly to regulate interspecies interactions. To survive, they end up crossing human boundaries (e.g., foraging refuses, nesting in buildings, trespassing properties), being consequently labelled as pests and disease vectors, messy scavengers, aggressive intruders, or a nuisance, and almost invariably removed (46, 47).

While displacing and marginalising wild animals, humans have also confined domesticated animals in segregated man-made environments, such as zoos, research laboratories or factory farms, where individuals are often severely constrained and have little control over their surroundings, and where they are unable to exercise agency to access resources and regulate social interactions (48), including implementing appropriate distance-setting mechanisms. As Calhoun's abovementioned experiments with rats demonstrated (12), when animals cannot maintain privacy boundaries as they want, their social organisation may become dysfunctional and their behaviours may become aberrant. More generally, it has been shown that, even when their physical needs are met, when animals are placed *in situations* that do not allow them to attain what they want (49, 50), their welfare can be severely compromised; this can lead to a deterioration of physical health, frequently resulting in the emergence of pathogens, and the spread of zoonotic bacterial and viral infections among animals and, indeed, humans.

In response to the segregation of animals, whether through displacement or confinement, some have called for multispecies integration. For example, instead of fighting back urban fauna, biologists Beatley and Bekoff (47) propose adjusting city planning policies and practises to integrate animal biodiversity into urban development and facilitate multispecies coexistence. This would include interventions such as planting and protecting autochthon vegetation, and creating animal-friendly passageways that allow animals to move around without encountering humans. At the same time, the authors propose increasing the visibility of and celebrating animals' presence in urban environments to increase the fascination and enjoyment that can derive from human-animal encounters (47). Consistent with this view, the work of urban architects, such as Metcalfe (51), has shown how it is possible to design environments that meet the needs of animal and human dwellers, thus facilitating multispecies coexistence. Designers of agricultural production systems, such as van Weeghel et al. (48), have also been advocating and experimenting with architectural and technological solutions that enable animals to take control over aspects of their living environment and production practises. Such measures allow animals to exercise agency and, at least a measure of, autonomy as active participants in production processes, aiming to improve their welfare.

These important initiatives aim to create more hospitable environments for animals, in which multiple species can coexist, and to support animals' agency, including their ability to manage their interactions with others. We suggest that animal privacy considerations should be part of these proposals, because privacy is essential for harmonious cohabitation and good individual and collective welfare. Furthermore, because animals' behaviour shows that their privacy matters to them, humans have an ethical responsibility to consider animals' privacy requirements when developing technological interventions that can impact on animals' ability to manage their privacy boundaries, thus jeopardising the effectiveness of their distance-setting mechanisms and preventing said mechanisms from fulfilling their biological function. While some of this responsibility might be fulfilled by enforcing existing animal protection laws or developing new such laws, laws are an expression of societal ethical values; thus, before the importance of animal privacy can be properly reflected into the law, it needs to be acknowledged as a societal ethical value. Consistent with this, we call for a fundamental consideration of animal privacy in the design of technological interventions and the ethical values they reflect. We suggest that an investigation of animals' species-specific distance-setting (including physical separation and information management) mechanisms should inform the requirements specification for the design and development of any technologically supported or enhanced environment in which animals are expected to dwel.

#### The Potential of Interactive Technology

Interactive technologies have a role to play in the realisation of interventions that could foster harmonious multispecies cohabitation, as well as individual and collective welfare. Thanks to their ability to respond to the actions of individuals and groups, to dynamically modify spaces, and to influence behaviour, interactive technology-integrating sensing and actuating mechanisms-arguably makes it at least plausible to create smart systems and environments that could account for animals' privacy requirements, balancing the needs of different stakeholders.

Interactive maps and augmented reality applications could be designed to educate the public about the privacy needs of animals living in cities or in particular areas of the countryside, and about the importance of respecting their privacy for welfare and conservation purposes. Human users could be encouraged to refrain from engaging in potentially intrusive or disruptive behaviours when resident animals are engaging in activities that require privacy. This might include, during mating or nursing periods, staying away from certain areas to allow animals physical space or keeping noise to a minimum to allow for the transmission of intimate communication signals. Such systems could also provide information to help users learn about the role of the species within the ecosystem, hopefully inspiring empathy and respect for non-human cohabitants.

Interactive technology could also be designed to enable animals to set privacy boundaries when they live within the constraints of captive environments. For example, in farms, kennels, zoos, and laboratory facilities, ambient sensors and intelligence might be used to recognise stress related to lack of privacy, similarly to the way in which ambient systems can now detect indicators of disease well ahead of an outbreak, based on collective behavioural patterns (52, 53). One could envisage a system of telescopic retractable partitions or roosts that was automatically activated when privacy-related stress was detected. These barriers and perches could dynamically change the configuration of a space to give resident animals temporary access to more private sections and levels, to provide secluded areas for individuals or small groups at particular times (e.g., during sleeping hours) while allowing free-flow circulation at other times.

Naturally, all such systems would need to be designed to protect the security of the data that they generate to help prevent ill-intentioned behaviours. For example, whenever individual animals were tracked and their activities recorded using wearable or ambient devices, data security would be essential to stop, for example, as poachers from accessing information that might facilitate their illegal practises. At the same time, mobile apps designed for legal practises that aim to raise awareness about animals' activities and their privacy needs could employ mechanisms, such as information “blurring,” to ensure that the animals' location or other sensitive information was not disclosed and, thus, prevent misuses of such systems, which might range from intentionally disturbing animals for curiosity to illegally culling them for personal interest. However, even perfectly legal and well-intentioned uses of such systems could have unexpected impacts that might actually exacerbate the already imbalanced relationship between humans and animals. For example, based on a system's suggestion, well-intentioned citizens might avoid frequenting a certain recreational area so as not to encroach on the resident animals during the breeding period, migrating to an alternative area instead; however, the increased influx to this other area might encroach on the resident humans, who might become hostile to the animals they see as the source cause of the inconvenience.

In this regard, van der Linden (54) argues for the importance of taking a holistic and systemic approach to the design of Interspecies Information Systems, analysing the possible interplay among humans, animals and technology in their sociotechnical context and how this may influence human behaviour toward animals. The author identifies key challenges for designers to consider-including how to understand the potential of animal data, how to effectively transform data into interspecies interventions, and how to assess the short and long term impacts of such interventions-stressing the importance of interdisciplinary collaboration to properly understand the requirements that interspecies information systems might need to meet, before they are deployed.

### The Paradox of Using Technology to Protect Animals' Privacy

We have seen how many species value their privacy, go to great lengths to manage their own privacy boundaries, and operate within ethics that recognise the privacy boundaries of others. On such grounds, we have argued that animal privacy should be an essential consideration when designing technological interventions and, further, that technologies could be developed specifically to protect animals' privacy or enable them to manage their privacy boundaries. However, it seems almost inevitable that such interventions would themselves intrude animals' privacy in order to provide the envisaged benefit of protecting it; this seems paradoxical, particularly if we assume that animals are unable to provide informed consent. Would it not be better to just leave animals alone instead of monitoring their activities, modifying their environment and gathering what could be regarded as their personal data? On the other hand, given humans' expansion proclivities, if we refrained from intruding animals' space with technological interventions and left them alone, would we not just continue to breach their privacy boundaries, whether intentionally or unintentionally? These are difficult but important questions, the answer to which is likely to depend on the particular context in which humans and animals live and operate. For instance, a fair use of monitoring technologies might help us to understand, recognise, and thus protect animals' privacy needs. But what constitutes “fair” use of such technologies is likely to depend, for example: on the kind of monitoring intervention envisaged in a particular setting; the potential impact or risk the monitoring activity might have for the animals involved; the vulnerability or endangered status of the species, group or individuals concerned; the availability of essential resources relative to human and animal population density, and the likelihood of interspecies frictions due to resource shortages. Arguably, animal stakeholders' perspective on what is “fair technology use” ought to be part of the equation.

In this regard, Mancini highlights the importance of garnering animals' consent when conducting research with them, on both ethical and scientific grounds (55). The author distinguishes between two forms of consent, highlighting the parallel with the forms of consent required when conducting research with children. *Mediated consent* would need to be provided by the humans who are legally responsible for the animals, know them well and have their best interest at heart, on the grounds that they are in a position to assess the wider welfare implications of the animals' involvement. However, animal participants themselves would need to provide *contingent consent*, as expressed by their willingness to engage with research set-ups and procedures, on the grounds that the animals are best placed to assess the immediate contingencies that make their involvement desirable for them. For the author, both forms of consent are necessary, because they reflect complementary capacities and equally important perspectives. Similarly, when determining what constitutes fair use of technology, the perspective mediated by humans on behalf of animals and the perspective of the animals themselves are equally important. In other words, humans might be able to determine that a temporary intrusion is in the long-term interest of the animals in question, but the animals themselves might be best placed to assess whether the intrusion is desirable given specific contingencies (this is especially important where technological interventions might impact, in the short or long term, animals' ability to fulfil fundamental needs-e.g., protecting their or their offsprings' safety, accessing vital resources-as opposed to expressing preferences that are not of vital importance). Particularly where the expected benefit of a technological intervention is the protection of their privacy and the enablement of their privacy management strategies, control over such interventions ought to be shared with animal stakeholders. In other words, animal stakeholders should be able to influence the behaviour of the technology and of its human users, such that systems' impact was *bi-directional*-to use a term proposed by van der Linden (54).

Thus, as far as possible, technological interventions could be designed to enable animals to asses said contingencies and to allow them to dissent, such that their dissent impacts the behaviour of the technology and of its human users. For example, mobile monitoring systems (e.g., robots, drones) might be designed to recognise the signs of animals' unease to their proximity and automatically retreat out of the way. What animals' dissent to physical intrusion might imply for the design of digitally intrusive interventions (e.g., hidden cameras and sensors collecting privacy-sensitive data) and how animals' privacy preferences might be enabled to influence such digital intrusions is to be explored, but the difficulty of imagining possible solutions should not prevent designers from asking this kind of question. Whatever the answers in specific circumstances, we suggest that it is important to ask these questions. Indeed, when Mills (8) questioned the ethics of filming animals in their private moments he was not arguing for a ban on such filming practises; rather, he was bringing to our attention the importance of not taking for granted the legitimacy of trespassing animals' boundaries. Behind the “right to be let alone,” advocated by Warren and Brandeis (10) and invoked by Aratym (9), is a fundamental universal need, and we propose that this universal need to be let alone should be part of the equation when designing any technological system that has the potential to affect animals.

## Conclusions

More than ever before, human activity is having a massive impact on other animals, destroying natural ecosystems and the species who inhabit them, while expanding artificial ecosystems in which billion of animals languish. Among the most fundamental animal needs that human practises are disregarding is privacy, all too often regarded as irrelevant when it comes to other-than-human species. In this paper, we have questioned this assumption. To this end, we have reviewed some of the ethological and behavioural experimental literature demonstrating that, to varying extents, animals manifest a broad range of behaviours to manage privacy boundaries, disclosing or concealing information (e.g., their presence, the presence of a resource, their intentions, their interests), through different mechanisms (e.g., physical separation or proximity, hiding from or sharing with, deception and disguise or openness) and channels (e.g., “confiding” vs “advertising)” in order to fulfil personal safety, sociality and intimacy, protecting assets, securing access to mates functions. In other words, privacy matters to animals and being able to manage their privacy boundaries is important for their survival. We therefore argue for the importance of accounting for animals' privacy requirements when designing interactive systems and technological interventions for, or that may affect, animals. In this regard, we discussed animal privacy as a design principle and explored the potential of privacy-aware systems to foster harmonious multispecies co-habitation and better animal welfare. By way of example, we have envisioned possible privacy-aware applications relevant to free-ranging and confined animals. More generally, we propose the notion of privacy-aware multispecies interaction design, and encourage interaction designers to apply their knowledge and skills to ensure that their work contributes to the development of a culture in which everyone's need to be let alone is respected for the benefit of all.

## Data Availability Statement

The original contributions presented in the study are included in the article/supplementary material, further inquiries can be directed to the corresponding author.

## Author Contributions

PP and CM contributed to the ideation and discussion of the argument proposed by the article, to the review of related literature and to the writing of its presentation, including the drafting of different sections and revisions of the overall draft. BN contributed to the ideation and discussion of the argument proposed by the article and to revisions of the overall draft. All authors contributed to the article and approved the submitted version.

## Funding

This work was supported in part by UK EPSRC grant EP/R013144/1 and SFI grant 13/RC/2094_P2.

## Conflict of Interest

The authors declare that the research was conducted in the absence of any commercial or financial relationships that could be construed as a potential conflict of interest.

## Publisher's Note

All claims expressed in this article are solely those of the authors and do not necessarily represent those of their affiliated organizations, or those of the publisher, the editors and the reviewers. Any product that may be evaluated in this article, or claim that may be made by its manufacturer, is not guaranteed or endorsed by the publisher.
